# Hyperdynamic ejection fraction in the critically ill patient

**DOI:** 10.1186/cc13369

**Published:** 2014-03-17

**Authors:** JR Paonessa, TP Brennan, LA Celi

**Affiliations:** 1 Beth Israel Deaconess Medical Center, Harvard Medical School, Boston, MA, USA; 2 Massachusetts Institute of Technology, Cambridge, MA, USA

## Introduction

The hyperdynamic left ventricular ejection fraction (HDLVEF) in the ICU is a common finding thought to be associated with critical illness and possibly sepsis. The exact etiology of hyperdynamic ejection fraction has yet to be determined, and the prognosis of these patients has not been well defined.

## Methods

The cohort consisted of 2,632 adults admitted to the ICU with echocardiogram reports using the MIMIC-II database, and was divided into those with HDLVEF and those with normal left ventricular ejection fraction (NLVEF). Those with impaired ejection fraction were excluded from the analysis. Baseline comparisons were performed using chisquared tests for equal proportion with results reported as numbers, percentages, and 95% CIs. Continuous variables were compared using *t *tests and reported as means with 95% CIs, while non-normally distributed data were compared using Wilcoxon rank-sum tests and reported as medians.

## Results

Patients with HDLVEF had increased mortality in hospital, at 28 days and at 1 year when compared with patients with NLVEF. HDLVEF patients more frequently required renal replacement therapy (RRT), vasopressors and mechanical ventilation. Of the 2,632 patients, 1,220 were septic. There was an increased proportion of HDLVEF in the septic compared with the nonseptic groups (11.2% vs. 8.6%, *P *= 0.026). Interestingly, other statistically significant associated comorbidities were cancer, CHF, arrhythmias, and hypertension, which were more commonly seen in the HDLVEF group. See Table 1.

**Table 1:** 

	Normal (*n *= 2,373, 90%)	Hyperdynamic (*n *= 259, 10%)	*P *value
Male	1,159 (49)	98 (38)	**<0.001**
Service type			
MICU	1,234 (52)	138 (53)	0.695
CCU	364 (15)	28 (11)	0.052
SICU	572 (24)	69 (27)	0.367
CSRU	188 (8)	23 (9)	0.590
Primary outcome			
Twenty-eight-day mortality	458 (19)	79 (31)	**<0.001**
One-year mortality	892 (38)	129 (50)	**<0.001**
ICU mortality	296 (12)	60 (23)	**<0.001**
Hospital mortality	433 (18)	80 (31)	**<0.001**
Treatment			
RRT	363 (15)	55 (21)	**0.013**
Vasopressor	1,063 (45)	139 (54)	**0.006**
Ventilatec	1,453 (61)	186 (72)	**<0.001**

## Conclusion

Patients with hyperdynamic LVEF in the ICU clearly have increased mortality. Hyperdynamic LVEF may be a result of increased catecholamines during cytokine storm. It is unclear whether hyperdynamic LVEF itself worsens outcomes. Further investigation is needed.

